# Benign Notochordal Cell Tumours: Case Report and Literature Review

**DOI:** 10.3390/diagnostics14131330

**Published:** 2024-06-23

**Authors:** Dagnija Grabovska, Ilze Strumfa, Janis Ositis, Inta Liepniece-Karele, Arturs Balodis

**Affiliations:** 1 Faculty of Residency, University of Latvia, LV-1586 Riga, Latvia; 2 Institute of Diagnostic Radiology, Pauls Stradins Clinical University Hospital, LV-1002 Riga, Latvia; 3 Department of Pathology, Riga Stradins University, 16 Dzirciema Street, LV-1007 Riga, Latvia; ilze.strumfa@rsu.lv (I.S.); inta.liepniece-karele@rsu.lv (I.L.-K.); 4 Department of Pediatric Surgery, Children’s Clinical University Hospital, LV-1004 Riga, Latvia; 5 Department of Radiology, Riga Stradins University, LV-1007 Riga, Latvia

**Keywords:** benign notochordal cell tumour (BNCT), chordoma, fibrous dysplasia, magnetic resonance imaging, differential diagnosis

## Abstract

Background: Benign notochordal cell tumours (BNCTs) represent a rare entity within the spectrum of bone neoplasms, which typically arise in the axial skeleton. Although these tumours are often benign, their diagnosis and management pose significant challenges due to their histological similarity to more aggressive lesions, such as chordomas. Understanding of the clinical behaviour, diagnostic nuances, and optimal management strategies for BNCTs continues to evolve. Case Report: Benign notochordal cell tumours of the vertebra are usually asymptomatic and identified on imaging and should be distinguished from chordomas, which has a more aggressive clinical course. This report describes a 15-year-old girl with lumbosacral pain and a diagnosis of a benign notochordal cell tumour, which affects a large part of the S1 vertebra in the lumbar spine, highlighting the diagnostic challenges encountered, the role of radiological and histological investigations, and the ultimate determination of the benign nature of the tumour. Conclusions: This report highlights the approach taken for the diagnosis of a benign notochordal cell tumour of the vertebra and the importance of excluding differential diagnoses. By exploring the intricacies of this case, we contribute to the growing body of literature surrounding BNCTs, with the aim of improving clinical awareness and management strategies for this uncommon bone tumour.

## 1. Introduction

The classification of notochordal tumours by the World Health Organization (WHO) includes benign notochordal cell tumours (BNCTs) and chordomas. Chordomas are rare tumours, with an incidence of 0.08 per 100,000 people [1]. The incidence of BNCTs is uncertain; their existence was documented more than a century ago and, following that, they were observed in the vertebrate column throughout the 20th century [1]. According to the WHO classification, BNCTs are the most frequent incidental findings during imaging studies conducted for unrelated reasons. The common sites of BNCTs have been reported to be the sacrum and clivus, but they are also often found in the cervical and lumbar regions of the spine. BNCTs show histological notochordal features, and they contain classical physaliphorous cells, but unlike chordomas, they lack cellular atypia, mitotic activity, an extracellular myxoid matrix, intratumoral vascularity, or necrosis [1,2]. Recently, due to the increased availability of MRI examinations, an increasing number of BNCT lesions have been discovered. Golden et al. conducted a study of 916 patients, using CT and MRI to examine the posterior clivus, and documented that the imaging prevalence of BNCTs was 0.76% [3]. Although BNCTs are generally considered benign, their histological characteristics can mimic more aggressive neoplasms, such as chordomas or chondrosarcomas, posing challenges in accurate diagnosis and treatment planning [4]. The common locations of BNCTs are similar to those of chordomas, and in rare cases, a BNCT has been reported to co-exist with a chordoma. Several reports propose the possibility that a BNCT can potentially act as a precursor lesion for a chordoma [5,6]. However, some researchers reject this hypothesis due to insufficient evidence [7]. Overall, the connection between chordomas and BNCTs is yet to be clarified [3].

This case report presents a unique case of a BNCT involving a large portion of the S1 vertebra in the lumbar spine in a 15-year-old girl, highlighting the diagnostic challenges encountered, the role of radiological and histological investigations, and the ultimate determination of the benign nature of the tumour. By exploring the intricacies of this case, we contribute to the growing body of literature surrounding BNCTs, with the aim of improving clinical awareness and management strategies for this uncommon bone tumour.

## 2. Case Report

A 15-year-old girl presented to the outpatient clinic with complaints of pain in the lumbar sacral region. A further radiological examination was scheduled to determine the cause of the pain.

**Figure 1:**
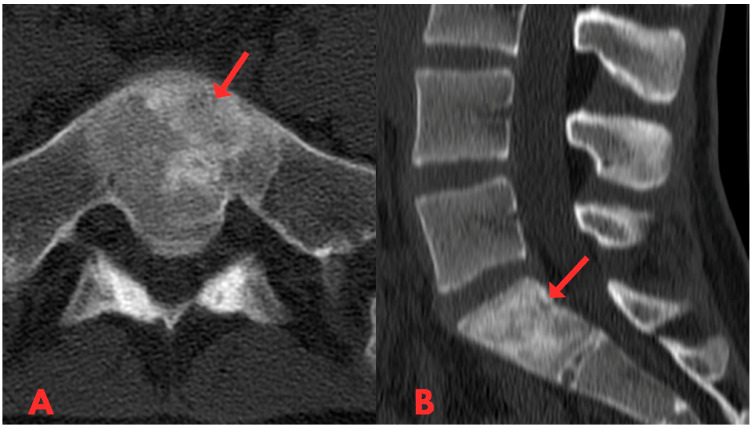
First CT examination of the lumbar spine axial (**A**) and sagittal (**B**) planes, showing heterogeneous sclerotic changes in the body of the S1 vertebra (red arrow).

Initially, a CT examination of the lumbar spine was performed, which revealed heterogeneous sclerotic changes in the body of the S1 vertebra (Figure 1). Interpretation suggested possible post-traumatic changes in the S1 vertebral body; however, since the patient had no history of trauma, this diagnosis was ruled out.

**Figure 2:**
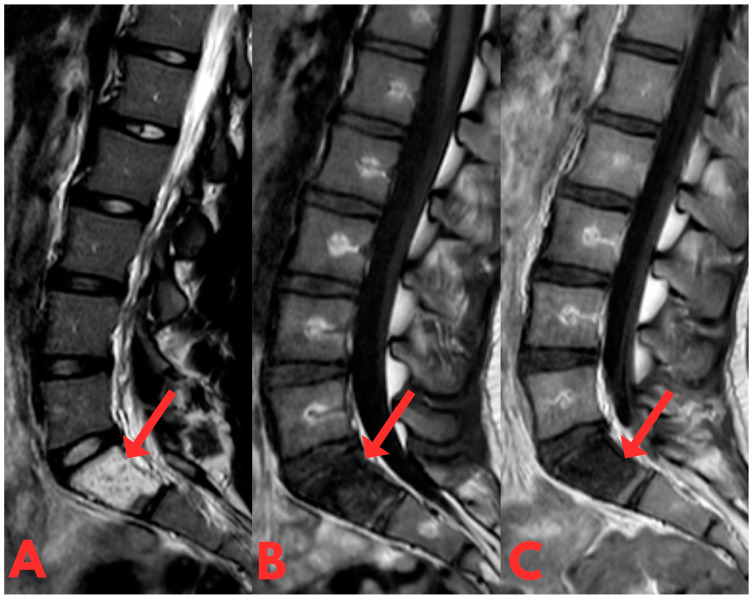
First T1- and T2-weighted sagittal contrast-enhanced MRI of the lumbar spine (T2W_TSE, T1W_TSE, T1W_TSE c+ sag) showing a sclerotic lesion with a hyperintense signal on T2 (**A**) and hypointense on T1 (**B**,**C**), which occupies practically the entire body of the S1 vertebra. Spread is not seen to the anterior epidural space or the prevertebral space.

Subsequently, a contrast-enhanced MRI examination of the lumbar spine revealed a centrally located sclerotic lesion with a hyperintense signal on T2 and hypointense on T1, measuring 2 × 4 cm in the corpus of the S1 vertebra (Figure 2). Imaging characteristics were indicative of fibrous dysplasia due to its characteristic features on MRI examination (T1: usually intermediate to low heterogeneous signal; T2: variable signal [8,9]), with a differential diagnosis of osteoid osteoma or another benign lesion.

**Figure 3:**
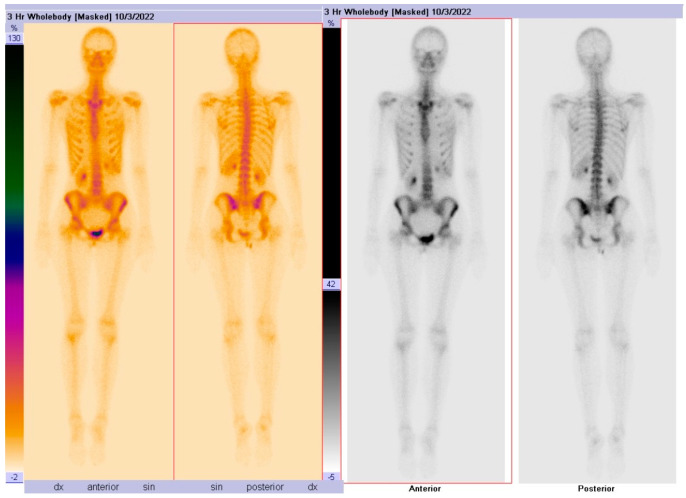
A static skeletal scintigraphy of the pelvic bones, which did not show conclusive evidence of pathological changes in the bones. No signs of osteoblastic lesions.

A static skeletal scintigraphy of the pelvic bones was performed, which did not show conclusive evidence of pathological or osteoblastic changes in the bones (Figure 3).

**Figure 4:**
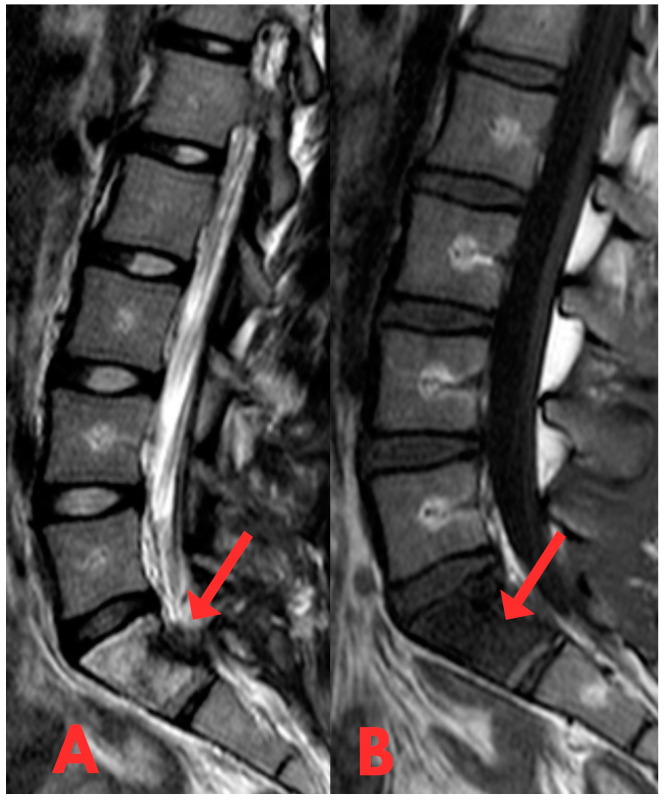
(**A**,**B**) Second T1- and T2-weighted sagittal MRI of the lumbar spine (T2W_TSE, T1W_TSE sag) showing no significant change in the S1 lesion. In dynamics, there is a small signal change in the posterior 1/3 of the S1 vertebra body after a biopsy with a small defect.

**Figure 5:**
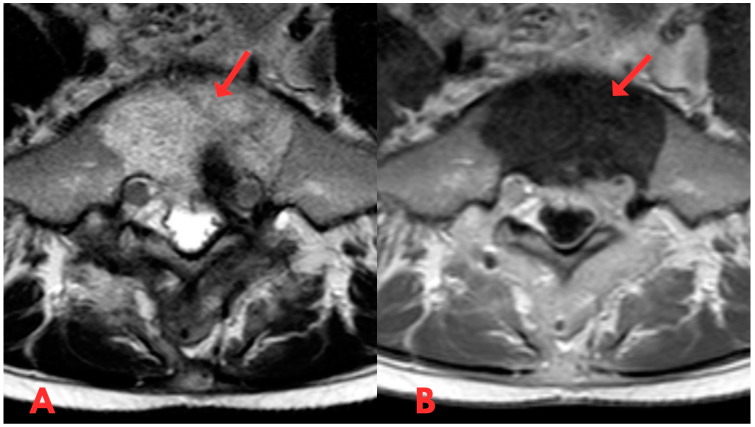
(**A**,**B**) Second T1- and T2-weighted axial MRI of the lumbar spine (T2W_TSE, T1W_TSE ax) showing an S1 vertebral lesion with a hyperintense signal on T2 (**A**) and hypointense on T1 (**B**) after a biopsy. Dynamics without change in the prevertebral and spinal canal.

The patient was neurologically intact but reported localised pain exacerbated by bending backward. No external abnormalities were observed and there was no relevant medical history of secondary illnesses or oncological diseases. A biopsy was performed with a 3.5 mm vertebroplasty needle. The obtained material was sent for histological examination. The histological response did not indicate significant findings. The diagnoses, fibrous dysplasia on MRI, and histologically small fragments of bone trabeculae did not provide a basis for a more surgically aggressive or conservative specific treatment method. Therefore, it was decided to perform a repeated open biopsy, to clarify the diagnosis and to examine the structure of the lesion under an operating microscope. A repeat MRI examination of the lumbar spine was performed, revealing a persistent high-signal lesion in the S1 vertebral body (Figure 4 and Figure 5), suggestive of fibrous dysplasia. Compared to the previous MRI, there were no changes in the dynamics of the radiological image.

**Figure 6:**
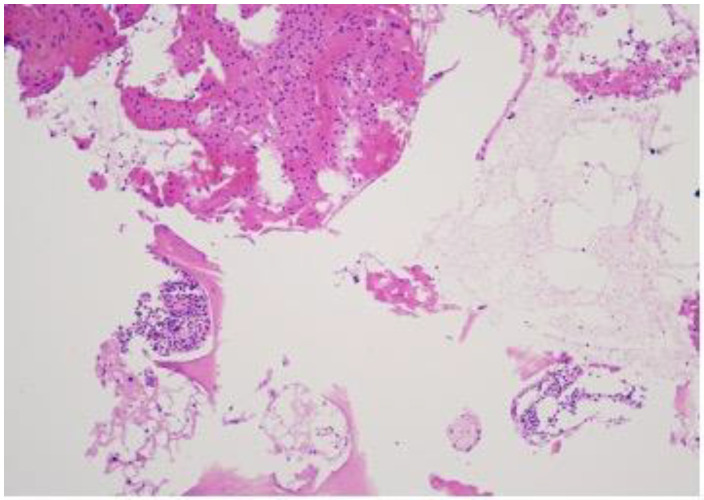
H&Ex10. A bone tumour consists of solid sheets of vacuolated cells with bland round nuclei.

**Figure 7:**
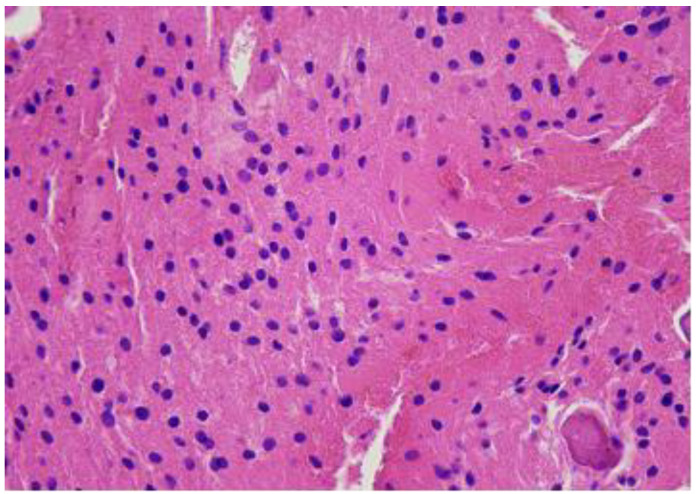
H&Ex20. A bone tumour consists of solid sheets of vacuolated cells with bland round nuclei. The tumour cells mimic mature fat or brown fat cells. No intracellular myxoid matrix, and no necrosis or mitotic figures.

**Figure 8:**
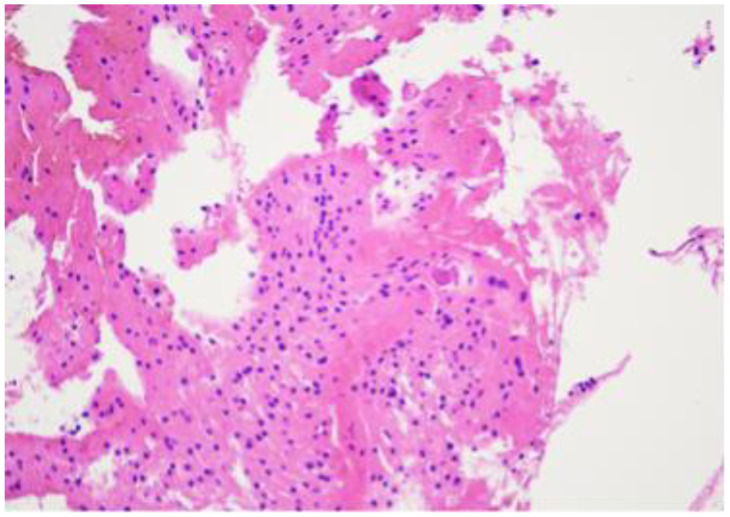
H&Ex10. A bone tumour consists of solid sheets of vacuolated cells with bland round nuclei. The tumour cells mimic mature fat or brown fat cells. No intracellular myxoid matrix, and no necrosis or mitotic figures.

A repeated biopsy of the lesion was performed, and histological analysis revealed finely calcified fragments and small bone trabecular elements in the biopsy material. A third control contrast-enhanced MRI examination of the lumbar spine was performed, which demonstrated a non-homogeneous hyperintense structure in the S1 vertebral body without contrast enhancement, extending into the spinal canal. Malignant bone tumours were ruled out and fibrous dysplasia was considered probable. It was decided to perform an open biopsy and S1 laminoplasty. The choice to perform S1 laminoplasty was based on the individual experience of the operating surgeon, as well as the age of the patient, and intended to provide greater access and better visual control for the biopsy. After a two-year examination period, the histological diagnosis was finally determined. Morphologically, the material corresponded to a benign notochordal cell tumour (BNCT) of the bone (Figure 6, Figure 7 and Figure 8). 

**Figure 9:**
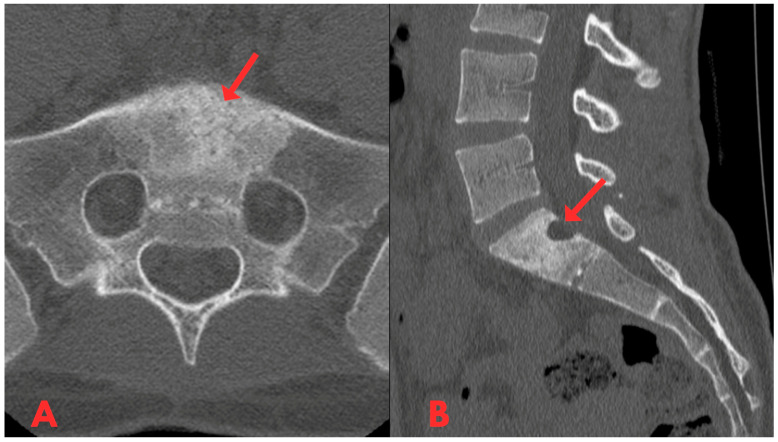
A follow-up CT examination of the lumbar spine axial and sagittal planes showing heterogeneous sclerotic changes in the body of the S1 vertebra, and compared to the previous CT and MRI scans, there were no significant changes in the dynamics of the radiological image. The posterior part of the S1 vertebral body shows a small defect after the biopsy.

A follow-up CT examination of the lumbar spine was performed (Figure 9), and compared to the previous CT and MRI scans, there were no significant changes in the dynamics of the radiological image.

## 3. Discussion

The presented case of a benign notochordal cell tumour (BNCT) highlights the diagnostic challenges and management considerations associated with this rare bone neoplasm. In the case presented, initial radiological investigations suggested fibrous dysplasia, and multiple rounds of imaging and biopsies were required to elucidate the true nature of the lesion. The diagnostic challenge lies in distinguishing BNCTs from their more aggressive counterparts, which requires a comprehensive evaluation combining radiological, histological, and clinical parameters. The differential diagnosis of BNCTs often includes chordomas, which are locally aggressive neoplasms with a predilection for the axial skeleton [1]. Histological analysis, an important part of the establishment of the diagnosis of a BNCT, often reveals the characteristic notochordal cell morphology. However, as seen in this case, histological features may not always be definitive, and ancillary techniques, such as immunohistochemistry, may be required to confirm the diagnosis [10]. The difficulties in the morphological diagnostics can be attributable to the focality and small size of the lesion, making it difficult to obtain a representative sample. Furthermore, BNCTs must be distinguished from chordomas, keeping in mind the possibility of a combined pathology with areas of BNCT and chordoma.

Morphologically, a BNCT is characterised by vacuolated cells featuring bland, round nuclei located at the periphery of neoplastic cells. Hence, the tissues bear a strong resemblance to adipocytes and/or multivacuolated brown fat cells. In contrast with chordomas, the extracellular myxoid matrix is absent [11,12]. Regarding the eosinophilic cellular component, Yamaguchi et al. noted the presence of this within the sheets of neoplastic cells [12], while Du et al. emphasised the absence of ribbons or cords of eosinophilic cells embedded in the extracellular myxoid matrix [11]. In summary, the eosinophilic component is present in a BNCT, but its architecture differs from that in a chordoma. The eosinophilic cells of a BNCT have a wide pink cytoplasm, contain few vacuoles, and may feature PAS-positive hyaline cytoplasmic globules. The nuclei in these cells are central, mildly enlarged, and can have a small nucleolus [12]. Some BNCTs include cystic spaces with homogeneous, eosinophilic, and colloid-like contents, which stain positively with PAS and Alcian blue [12]. This feature must be promptly distinguished from the intercellular myxoid matrix in a chordoma. A BNCT lacks anaplastic features or mitotic activity. In contrast to a chordoma, a BNCT is not osteolytic. However, well-demarcated BNCT lesions are not encapsulated and thus can grow between bone trabeculae or push bone marrow with a sharp border [11,12]. The adjacent bone trabeculae can show sclerotic changes and appositional growth of new bone. Entrapped islands of bone marrow are frequent in a BNCT, and the tumour can even extend through the bone cortex, albeit rarely [12].

Immunohistochemically, BNCTs uniformly co-express cytokeratin AE1/3 (100%), epithelial membrane antigen (EMA) (100%, albeit focal in 7.7% of cases), and vimentin (100%) [12]. The invariable presence of S-100 protein (100%) compromises the differential diagnosis with other S-100-positive lesions of a similar morphology: adipocytes, lipoma, liposarcoma, lipoastrocytoma, glial tumours, chondroma, and chondrosarcoma, as well as chordoma. The expression of S-100 can be focal, as observed in 23.1% of BNCT cases. Cytokeratin 8/18 is most often present (92.3%) [11]. The co-expression of epithelial markers and S-100 reflects the notochordal origin [13]. Furthermore, expression of the nuclear transcription factor brachyury is characteristic in BNCTs. According to the WHO, brachyury is considered the diagnostic hallmark of a chordoma [14] because of its frequent occurrence (up to 79/80 chordoma cases, according to Miettinen et al., 2015 [15]) and high specificity in distinguishing a chordoma from morphological mimics such as a chondrosarcoma and mucinous adenocarcinoma [13]. Brachyury is present in the nuclei of BNCTs (100%) as well, but focal expression was noted in most cases (61.5%) by Du et al. [11]. In agreement with the findings on the focality of brachyury in BNCTs, Shen et al., when studying the origin of chordomas from foetal notochordal cell rests, reported that brachyury was positive in chordomas but absent from BNCT components adjacent to chordomas [16]. Phosphorylated p70S6 kinase and p-mechanistic target of rapamycin (p-mTOR) have been found in 84.6% and 76.9% cases of BNCTs, respectively, paralleling the upregulation of the m-TOR pathway in chordomas [12]. EGFR protein is rarely detectable by immunohistochemistry (7.7%), although through fluorescent in situ hybridisation (FISH), EGFR amplification has been highlighted in up to 30% of cases [11]. Moreover, CD24 can be found (53.8%). Typically, the proliferation fraction by Ki-67 is low, mostly described as below 1%, 2%, or 3%. It does not exceed 1% in most (61.5%) cases, but in some series, up to 23.1% of BNCTs have shown a proliferation fraction of 3% [11]. When considering chromosomal changes, copy number gain is common in BNCTs, in contrast to more frequent copy number losses in chordomas [11].

Radiologically, BNCTs often have distinctive features that help in their characterisation, although challenges in differentiation from other bone neoplasms persist. The case at hand, supported by various imaging modalities, presents the typical radiological characteristics associated with BNCTs. Computed tomography (CT) scans may reveal sclerotic changes within the lesion, contributing to the differential diagnosis [17]. In some cases, as seen in our patient’s initial CT scan, heterogeneous sclerotic changes in the body of the S1 vertebra may be evident. However, CT alone may not be sufficient for a definitive diagnosis due to the overlap of imaging features with other bone tumours. The primary imaging modality for a BNCT is magnetic resonance imaging (MRI). A BNCT typically manifests as a well-defined lesion with low to intermediate signal intensity in T1-weighted images and high signal intensity in T2-weighted images. The characteristic hyperintensity in T2-weighted images can help distinguish BNCTs from more aggressive entities such as chordomas [17,18]. The characteristic feature of BNCTs in contrast-enhanced MRI is a lack of significant enhancement [17]. In our case, persistent high signal intensity on repeat MRI and lack of significant enhancement supported our suspicion of fibrous dysplasia. This contrasts with malignancies, where marked enhancement is often observed [17]. The absence of contrast enhancement, cortical permeation, bone destruction, or soft tissue components in the presented case supported the consideration of a benign pathology [17,18]. The distinctive radiological characteristics observed in our case align with previous reports in the literature, emphasising the utility of MRI to guide the diagnosis of a BNCT. However, it is crucial to acknowledge that while typical characteristics exist, a definitive diagnosis often requires histological confirmation, as demonstrated by the subsequent biopsies and surgical procedures in our case. Nonetheless, the radiological evaluation of a BNCT plays a pivotal role in the diagnostic process. The typical features observed in MRI and CT studies contribute to a preliminary characterisation, guide further investigations, and ultimately aid in the multidisciplinary approach required for an accurate diagnosis and optimal patient management. The management of BNCTs remains conservative due to their benign nature, but surgical intervention is considered when the tumour causes symptoms or presents diagnostic uncertainties [3,10], as demonstrated in this case, where a biopsy and laminoplasty were performed for the definitive diagnosis and treatment planning.

## 4. Conclusions

In summary, this case report of a benign notochordal cell tumour (BNCT) has highlighted the approach taken for the diagnosis of a benign notochordal cell tumour of the vertebra and the importance of excluding differential diagnoses, and it contributes to the growing body of literature on BNCTs, emphasising the need for a multidisciplinary approach to accurately diagnose these tumours and ensure optimal patient management. Further collaborative efforts and the reporting of similar cases will advance our understanding of the clinical behaviour of BNCTs and refine the management strategies for this rare bone tumour.

## Data Availability

The data presented in this study are available on request from the corresponding author.
